# NAD^+^ restores proteostasis through splicing-dependent autophagy

**DOI:** 10.1080/15548627.2025.2596679

**Published:** 2025-12-29

**Authors:** Ruixue Ai, Evandro F. Fang

**Affiliations:** a Department of Clinical Molecular Biology, University of Oslo and Akershus University Hospital, Oslo, Lørenskog, Norway; b The Norwegian Centre on Healthy Ageing (NO-Age) and the Norwegian National Anti-Alzheimer’s Disease (NO-AD) Networks, Oslo, Norway

**Keywords:** Aging, alzheimer disease, machine learning, NAD^+^ precursors, rna splicing, tauopathy

## Abstract

Autophagy preserves neuronal integrity by clearing damaged proteins and organelles, but its efficiency declines with aging and neurodegeneration. Depletion of the oxidized form of nicotinamide adenine dinucleotide (NAD^+^) is a hallmark of this decline, yet how metabolic restoration enhances autophagic control has remained obscure. Meanwhile, alternative RNA splicing errors accumulate in aging brains, compromising proteostasis. Here, we identify a metabolic – transcriptional mechanism linking NAD^+^ metabolism to autophagic proteostasis through the NAD^+^ -EVA1C axis. Cross-species analyses in *C. elegans*, mice, and human samples reveal that NAD^+^ supplementation corrects hundreds of age- or Alzheimer-associated splicing errors, notably restoring balanced expression of EVA1C isoforms. Loss of EVA1C impairs the memory and proteostatic benefits of NAD^+^, underscoring its essential role in neuronal resilience. Mechanistically, NAD^+^ rebalances EVA1C isoforms that interact with chaperones BAG1 and HSPA/HSP70, reinforcing their network to facilitate chaperone-assisted selective macroautophagy and proteasomal degradation of misfolded proteins such as MAPT/tau. Thus, NAD^+^ restoration coordinates RNA splicing fidelity with downstream proteostatic systems, establishing a metabolic – transcriptional checkpoint for neuronal quality control. This finding expands the paradigm of autophagy regulation, positioning metabolic splice-switching as a crucial mechanism to maintain proteostasis and suggesting new strategies to combat aging-related neurodegenerative diseases.

## Metabolic cues as regulators of autophagy fidelity

Autophagy safeguards neurons by eliminating dysfunctional proteins and organelles, but its capacity declines with age and especially in neurodegenerative diseases such as Alzheimer disease (AD). A major contributor to this decline is waning levels of the oxidized form of nicotinamide adenine dinucleotide (NAD^+^), a central metabolic cofactor essential for mitochondrial function, DNA repair, and stress resilience. While NAD^+^ depletion is recognized as a cause (or at least risk factor) of aging and neurodegeneration, how metabolic restoration translates into improved autophagic control has remained unclear.

In parallel, compromised alternative RNA splicing events (ASEs) have emerged as another layer of neuronal vulnerability. Splicing errors accumulate during aging and in tauopathies, altering the expression of proteins crucial for synaptic, mitochondrial, and proteostatic function. Yet whether metabolism communicates with the splicing machinery, and how this interaction affects autophagy, has been unexplored.

We recently demonstrated that NAD^+^ supplementation restores ASEs and proteostasis through the previously unrecognized NAD^+^ -EVA1C axis, establishing a mechanistic bridge between metabolism, ASEs, and autophagy [1].

## NAD^+^ restores defective ASEs through EVA1C

Cross-species analyses in *C. elegans*, mouse, and human systems revealed that NAD^+^ augmentation corrects hundreds of aberrant ASEs associated with aging and AD. Among these, EVA1C (eva-1 homolog C) emerged as a key NAD^+^ -responsive gene whose splicing pattern and isoform balance are selectively normalized by NAD^+^ precursors.

EVA1C belongs to the immunoglobulin superfamily and is enriched in neurons, where it contributes to axonal development and guidance. In human brains, EVA1C protein levels are markedly reduced in early Braak stages, suggesting its decline is an early molecular signature of AD pathology. Functionally, hippocampal knockdown of *Eva1c* abolishes the memory improvement induced by NAD^+^ supplementation in tauopathy mouse and worm models, demonstrating that EVA1C is essential for the cognitive and proteostatic benefits of NAD^+^.

These findings position NAD^+^ -regulated ASEs as a critical determinant of neuronal resilience, acting through precise control of EVA1C isoform expression.

## EVA1C couples rna splicing fidelity to autophagic proteostasis

While EVA1C does not directly regulate RNA splicing, it functions as a downstream translator of splicing fidelity into protein homeostasis. Structural modeling and protein-protein interaction predictions revealed that specific EVA1C isoforms differentially associate with the chaperone proteins BAG1 and HSPA/HSP70, both core regulators of autophagy and the proteasome system.

In human cell and mouse models of tauopathy, NAD^+^ supplementation increases BAG1 and HSPA/HSP70 abundance and strengthens their interaction with EVA1C. This coordination defines a cascade:

NAD^+^ restoration → correction of RNA splicing → EVA1C isoform rebalancing → BAG1-HSPA/HSP70 activation → enhanced autophagic proteostasis.

**Figure 1.**
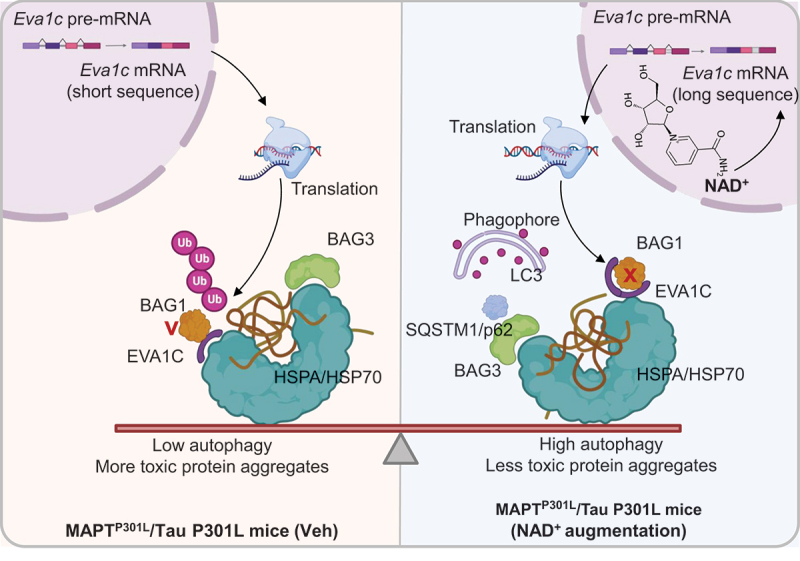
NAD^+^ -regulated isoform switching of EVA1C redirects protein quality control from proteasomal to autophagic degradation in tauopathy. Under pathological MAPT^P301L^/Tau (P301L) conditions, *Eva1c* pre-mRNA is predominantly spliced into its short isoform, which preferentially binds HSPA/HSP70 to promote proteasomal degradation of polyubiquitinated proteins, maintaining basal protein quality control. NAD^+^ elevation, however, shifts *Eva1c* splicing toward the long isoform through alternative RNA splicing. The long EVA1C preferentially associates with BAG1, thereby switching the degradation pathway from the proteasome to selective macroautophagy, i.e., chaperone-assisted selective autophagy/CASA. This NAD^+^ -dependent isoform conversion provides a mechanistic link between metabolic state and proteostasis, suggesting that boosting NAD^+^ remodels cellular clearance capacity by rewiring the HSPA/HSP70-BAG chaperone network toward autophagic MAPT/tau elimination.

In AD, reduced EVA1C expression coincides with early deficits in autophagy markers, supporting its role in maintaining neuronal quality control. Mechanistically, restored EVA1C isoforms may stabilize BAG1-HSPA/HSP70 complexes involved in chaperone-assisted selective autophagy/CASA (different from that of chaperone-mediated autophagy (CMA) which is an independent type of autophagy where specific cytosolic KFERQ-like motif-containing proteins are directly transported across the lysosomal membrane via chaperone HSC70 for lysosomal degradation). BAG1 links HSPA/HSP70-bound substrates to the proteasome and autophagic membranes, while HSPA/HSP70 governs substrate recognition and delivery to lysosomes. By reinforcing this chaperone network, NAD^+^ indirectly promotes autophagic clearance of misfolded MAPT/tau and restores proteostasis (Figure 1).

## A metabolic – transcriptional checkpoint for neuronal quality control

The discovery of the NAD^+^ -EVA1C-BAG1-HSPA/HSP70 pathway reveals a previously unrecognized mechanism that integrates metabolic sensing, RNA processing, and autophagy. By reprogramming splicing fidelity, NAD^+^ remodels downstream proteostatic networks and aligns nuclear RNA quality control with cytoplasmic degradation systems. This mechanism extends the role of NAD^+^ beyond mitochondrial metabolism and positions it as a systemic regulator of autophagy via RNA biology.

Rather than acting independently, metabolism and RNA splicing now appear to be metabolically synchronized layers of the same proteostasis network. The loss of NAD^+^ with age thus disrupts this synchronicity, weakening both RNA processing and autophagic clearance, and predisposing neurons to MAPT/tau aggregation and degeneration.

## Perspective

This work expands the conceptual landscape of autophagy regulation. It highlights ASEs as a previously overlooked determinant of autophagic capacity and introduces metabolic splice-switching as a new principle for sustaining proteostasis during aging. More detailed mechanisms on how EVA1C regulates autophagy (including how it physically interacts with BAG1 and HSPA/HSP70) are needed. Future studies should examine whether other splicing-sensitive proteins form similar metabolic checkpoints that link nuclear transcript integrity to cytoplasmic autophagic flux. Clarifying these connections may uncover new opportunities to reinforce neuronal homeostasis through combined metabolic and autophagy-targeted interventions.

## Data Availability

No new data were generated or analyzed in support of this publication.
